# COVID labour: Making a ‘livable’ life under lockdown

**DOI:** 10.1177/00380261221138203

**Published:** 2023-01

**Authors:** Katherine Twamley, Charlotte Faircloth, Humera Iqbal

**Affiliations:** Social Research Institute, University College London, UK

**Keywords:** cognitive labour, everyday life, families, pandemic, risk, trust

## Abstract

Drawing on qualitative longitudinal data from 38 families with children in the UK collected between May 2020 and June 2021, this article discusses the extra everyday labour which individuals experienced in going about their daily lives during COVID-19. In particular, we examine in detail the everyday practices of negotiating risk and caring for self and others within the context of the pandemic. We call this COVID labour – the *work* involved in living through and adjusting to a pandemic. We identify this as constituting three main aspects: seeking and interpreting information; assessing risk; and minimising risk. Like other forms of labour, it is stratified by gender, class and ethnicity. Overall, the analysis contributes to a greater understanding of everyday life ‘under lockdown’ for families with children, and how ‘livable’ lives are made under times of great risk.

## Introduction

In this article, based upon our in-depth longitudinal qualitative study, we detail the everyday labour that families with children take part in as they respond to and manage risks posed during the COVID-19 pandemic. We examine how people react to and negotiate government social distancing guidelines, and the ways in which these guidelines shape participants’ experiences. These analyses help us understand how families make a ‘livable’ life (Back, 2015) while attempting to keep themselves and those around them safe. Our data collection, undertaken between May 2020 and June 2021 in the UK, traces the adaptations and responses of families to a quickly changing context. In May 2020, for example, a ‘stay at home’ order was in place allowing for only minimal excursions from the home except for so-called ‘key workers’ who were necessary for the running of essential in-person services (such as medics, shop assistants and refuse collectors). Schools and childcare facilities, non-essential shops and leisure facilities were all shut or had moved online. Over the course of the following year, restrictions were partially and/or fully lifted and then reinstated again, sometimes with very late notice, as rates of infection and death rose and fell.^1^ As Deborah Lupton writes (2021), our participants were living in a ‘COVID Society’ (a spin on Beck’s ‘Risk Society’ thesis) where (consciousness of) risk and state intervention in everyday lives vastly increased, and where uncertainty was rife.

Our focus on families with children was driven by our desire to examine the additional demands placed on parents during the pandemic, and how they managed these. As other studies have recognised, parents’ (and particularly mothers’) domestic and care responsibilities hugely expanded when childcare and educational settings shut (Andrew et al., 2020). Less attention has been paid to how families negotiated and managed the *risks* associated with the pandemic. But notions of risk are deeply implicated in family life, worked out interdependently with others and connected to caring for others and oneself (Lupton, 2013). In this article, we examine in detail the everyday labour of negotiating risk and caring for self and others within the context of the pandemic. In describing this labour, our aim is to throw light on the cognitive load (Mullainathan & Shafir, 2013; Vohs et al., 2008) which underlay the shift to a context of heightened risk, and how the social positioning of participants shaped its manifestation.

The result is what we call COVID labour – the *work* involved in living through and adjusting to a pandemic or similar risk-laden upheavel.^2^ COVID labour can be understood as an intermediary domain between government guidelines and participants’ efforts to negotiate this new and uncertain landscape. Like other forms of domestic and care labour, it is gendered, but also, importantly, stratified by social class. Drawing on scholarship around ‘risk work’ (Brown & Gale, 2018; Gale et al., 2016) in unpacking the details of this labour, we identify COVID labour as constituting of three main aspects: seeking and interpreting information; assessing risk; and minimising risk. The analysis contributes to a greater understanding of everyday life ‘under lockdown’ for families with children, and how ‘livable’ lives are made under times of great risk, inculcating further but mitigating other kinds of risk.

## Living under lockdown in the UK

On 23 March 2020 the UK went into the first of three national lockdowns. Although the four nations of the UK set their own policies in relation to public health responses, there was mostly convergence, particularly during the first lockdown when all schools and childcare settings were closed except for the children of ‘key workers’ in essential services. A ‘stay-at-home order’ was introduced, with a ban on all non-essential travel and contact with people from outside one’s household. A gradual reopening of schools and childcare providers started in June 2020, with a full opening in September. A second lockdown was initiated in November 2020 for one month in most parts of the UK, but with schools and childcare facilities remaining open. Restrictions mainly centred around leisure facilities (such as restaurants and ‘non-essential’ shops). The third lockdown occurred in January 2021 for three months, on the back of local lockdowns and restrictions on inter-household mixing initiated immediately prior to Christmas. Once again schools moved online for all but the children of key workers, though nurseries remained open.

Several studies have analysed the increased care labour which was experienced by families provoked by the closing of childcare institutions. A time-use study found that in the first UK national lockdown, parents were doing childcare during nine hours of the day, and housework during three (Andrew et al., 2020). A special edition of *Gender & Society* (Mooi-Reci & Risman, 2021) unpacked the consequences of closures on men and women’s care work and ability to maintain paid employment. There is no doubt such events have led to vast increases in labour and in many cases expanded gendered inequalities. This was particularly acute for families living across households – for example, we had separated and living apart together (LAT) parents in our study who were initially unable to share care across households. In the first lockdown, children from these families did not see their fathers at all or only very briefly. In September 2020, the government introduced ‘bubbles’, whereby single parent and/or people in vulnerable households could legally mix.^3^

More generally, parents and children had to negotiate issues which were unproblematic or non-existent pre-pandemic, whilst isolation and confinement to the home disrupted previously taken-for-granted family routines and rituals (Prime et al., 2020). The combination of economic pressures, greater demands on parents’ time and resources, and reduced parenting capacity increased stress levels and the risk of turning to less constructive parenting strategies and harsh parenting, leading to tensions, conflict escalation and poorer relationships (Brown et al., 2020; Prime et al., 2020). Women and parents of young children reported particularly high levels of stress (Pierce et al., 2020). In many cases, however, family relationships also served as a buffer: close relationships can have a protective effect and family belief systems may foster ‘resilience’ or an increased sense of wellbeing (Prime et al., 2020); indeed, some families reported positive effects of spending more time together at home (Brown et al., 2020; McNeily & Reece, 2020; Neece et al., 2020).

The pandemic occurred in the UK at a time of increasing rates of inequality and poor funding of health and social care after years of ‘austerity’ policies. As Wood and Skeggs argue (2020), this has been part of an overall strategy by Conservative led governments towards the privatisation of health and care, thereby increasing personal (and profit-oriented) responsibility for the sick and elderly. Following this logic, while the state has taken a primary role in managing the response, it has done so in ways which have foregrounded personal responsibility (Preston & Firth, 2020; Williams, 2021). Government guidance frequently emphasised the role of individuals in making appropriate ‘choices’ in responding to various public health measures (Williams, 2021). And while police were given powers to enforce the lockdown, individuals were also encouraged to report observed infractions of their neighbours.^4^ As argued by Reicher et al. (2021), this approach encourages victim blaming, whereby those infected with COVID are characterised as responsible for their own infection and illness, while also deflecting attention away from government policies and their (in)effectiveness in managing the pandemic.

Nonetheless, overall adherence to public health measures as reported in surveys was found to be relatively high throughout the pandemic (Atchison et al., 2020; Smith et al., 2020). Young people, men, those of a minority ethnic background and those living in more deprived areas of the UK were least likely to adhere to public health measures, including in taking up the vaccine (Levita et al., 2020; Office for National Statistics [ONS], 2022). Several studies have explored the factors associated with variance in take up of public health measures. These studies show that individuals balance the risk of catching COVID with other perceived risks or needs, such as the need for social connection for their mental health, and financial concerns and responsibilities (Denford et al., 2021). Distrust in government sources, ‘alert fatigue’ and the impact of the vaccine on risk perception also impacted on take up (Ogueji et al., 2021; Williams et al., 2021). These studies show the importance of risk evaluation in determining behaviour, and how these evaluations differed across different groups and time periods. What is lacking is a more in-depth examination of the everyday practices which such deliberations involved and how this impacted on family life.

## Theoretical framework

In studying families’ lives during COVID-19, we draw on scholarship around everyday life, risk and parenting. First, in theorising the links between micro practices and wider social change we draw on scholarship from the sociology of everyday life. As Neal and Murji (2015, p. 812) comment, ‘Everyday life can be thought of as providing the sites and moments of translation and adaption. It is the landscape in which the social gets to be made – and unmade.’ Our focus is on how everyday life shifted for families with children in response to government COVID-related guidelines and a ‘COVID Society’ rife with risk (Lupton, 2021). Comparing the current context to that in which Ulrich Beck (1992) wrote *Risk Society*, Lupton (2021) posits that such processes observed by Beck in the 1990s may be further heightened during the COVID-19 pandemic. Beck argued that industrialisation and globalisation increased the scale and potential for catastrophic events. The heightened awareness of such risks framed social life, shaping ideas of selfhood, social relations and social institutions. In particular, he argued that faith in ‘experts’ and science was eroded while social institutions were no longer trusted to keep people safe. This sense of insecurity and lack of trust heightened individuals’ sense of personal responsibility in responding to and mitigating risks. As argued above, the particular framing of UK government public health guidance as dependent on the appropriate choices of individuals is likely to have further exacerbated the responsibilisation of our participants.

We draw on literature around risk work and cognitive labour in theorising the labour which our participants describe in responding to risk during the COVID-19 pandemic. Risk work is a concept from Brown and Gale, developed in analysing ‘the everyday experiences and practices of (para)professionals where risk has become a key and in some cases (re)defining feature of everyday work’ (2018, p. 1). The parallels are clear. While healthcare professionals are caring for patients at risk, our participants are caring for their families and themselves, adding a further relational dimension to their experiences (Twamley et al., 2021). ‘Risk work’ draws attention to the ‘practices which enable this work to “get done”’ and is threefold: interpreting risk knowledge, intervening to minimise risk, and handling social relations and interactions (Brown & Gale, 2018, p. 3). Cognitive or mental labour (or the ‘mental load’), meanwhile, comes from literature around parenting and recognises the work of ‘anticipating needs, identifying options for filling them, making decisions, and monitoring progress’ that is a part of family life (Daminger, 2019, p. 609; see also Yun-Suk & Waite, 2005). It is commonly overlooked in studies of family care and practice but is key to understanding gendered divisions of paid and unpaid work (Daminger, 2019). Women are usually found to do more cognitive labour than men in the domestic sphere, and more generally to take responsibility for managing risk within families (e.g. Mackendrick, 2014; Umamaheswar & Tan, 2020).

Scholarship in the field of Parenting Culture Studies brings together insights from each of these perspectives, to make the argument that the job of raising children has become a hugely expanded task in recent decades (Lee et al., 2014). Minimising risk to, and optimising development of, the child might be said to be two of the defining features of contemporary parenting culture (Hays, 1996). This has a particular moral resonance for mothers for whom messages about ‘good parenting’ are targeted and internalised (Lee et al., 2014). Much research in this field has pointed to the damaging influence of the individualised approach to caring for children and the idealised portrayals of a more ‘intensive’ motherhood, including the detrimental effects to the wellbeing of mothers (Hays, 1996; Rizzo et al., 2013).

Thus, our research focuses on families with children in the first year of the pandemic, as they manage risk to an unprecedented level. We outline the everyday practices which parents engage as they manage and adapt to risk, and the ways in which these were shaped by participants’ material resources and attempts to make a ‘livable life’ (Back, 2015). Here we are inspired by Les Back’s ethnographic study of festive lighting in a working-class area of England, where he reveals the importance of attending to the ‘complex structure of feeling with networks of interaction as well as structural dimensions’ (p. 833) in understanding the rituals and practices of people living in the midst of social damage. A ‘livable life’ during a pandemic has clear relevance, touching on both a sense of fulfilment but also the more basic task of staying alive. Following Back, therefore, we attend to everyday practices, as well as the emotional and meaning-laden ideas which underpin these practices. The result is ‘COVID labour’, which we describe in more depth below.

## Methods

This is a mixed-methods longitudinal comparative study. Data collection started in May 2020 and ended in June 2021. We recruited 38 families with children living in various parts of the UK through a short recruitment survey distributed via social media and outreach organisations. Not all families lived permanently within the same household, as we had some children and grandchildren living across or in separate households. All family member participants are given the same pseudonym to make their connection clear.^5^ Eleven families reported a household income of over £90,000 per annum; 14 between £30,000 and £90,000; and 13 less than £30,000.^6^ These incomes are based on parents’ estimations (therefore, grandparents who live separately are not included in this ‘household’ income). Ten of the families had at least one parent who was a key worker.^7^ Children in participant families ranged in age from 5 months to 17 years old. There were an average of 1.7 children in each household at the beginning of the study (three babies were born over the course of the study). The mean age of the children was 8 years old.

Everyone in the family age 12 and upwards was invited to participate in the study, but parents were also asked to reflect on children’s experiences of the pandemic (reported elsewhere). Overall, we had 73 individuals participating: 13 young people, eight grandparents and 52 parents. We focus on the adults in this article. Eighteen of the 60 adults come from a visible minority ethnic background: six identified as Black; 10 as of South Asian origin; one Chinese; and one from a mixed racial background. A high proportion of the adults had a university education (71%).^8^ Thirty-five of the parents were mothers, and seven of these single mothers. Given that most of our sample are women and that these women were the most responsive in our study, we draw only tentative conclusions about gendered differences in the experiences of COVID labour.

Most adult participants (36) completed multimodal diaries over the course of the study, with a final family level online interview in May/June 2021. Nine of these also completed an online individual interview in June 2020. For the diaries we used a data collection application (https://indeemo.com/) that facilitates entries via text, video and photos. Researchers can respond to these entries via follow-on questions or queries. These ‘mobile methods’ facilitate the collection of data *in situ* and increase the temporal immediacy of self-reporting, as participants receive a ‘text’ each time a new diary probe or question is uploaded (Boase & Humphries, 2018). Approximately 900 photographs with captions, 452 videos and 903 text posts were uploaded by participants, with the most intensive activity occurring in the initial four months. Sixteen participants took part via interviews only – an individual interview in June 2020 and a family level interview in June 2021.^9^ Lower income participants were more likely to choose to participate via video or telephone interviews only. Diary probes and interview questions were similar: we asked participants about their daily lives; how they stayed in touch with friends and family; sources of and responses to information about the pandemic; and when and why they ‘broke’ social distancing guidelines. Overall, we had similar findings from the different methodological approaches, but breaking of social-distancing guidelines was more often reported in diaries than in interviews. The different types of data (images, interviews and diary entries) were transcribed and analysed using thematic analysis techniques (Braun & Clarke, 2006) on NVivo. Our coding was both inductive and deductive, in that we applied theoretical codes from our literature review, while also generating new codes directly from the data. Codes for ‘COVID labour’ were inductively generated. We then used matrix tabulations for comparisons across groups.

Ethical approval was granted by the authors’ university ethics board. We were mindful in designing our study of the potential anxiety and stress which participants were likely to be experiencing, and respondents were regularly reminded of their ability to skip questions and activities, or indeed to drop out completely. The multiple methods described above were initiated as part of an effort to give our participants greater flexibility in how they participated (see Faircloth et al., 2022 for more details).

## What is COVID labour?

While sociologists note how the everyday ‘banal’ practices of individuals often go unnoticed, the pandemic has crystallised and refocused individuals’ sense of the everyday since ‘normal’ everyday practice has been disrupted and transformed. As Scambler (2020, p. 140) argues, the pandemic has functioned as a ‘breaching experiment’ which can provide us, as sociologists, ‘rare insights into the day-to-day practices, or artful accomplishment, of ordered living’. In this article, drawing on the rich qualitative data generated by the project, we focus on the transitioning experiences of families as they adapted to the pandemic and in particular with how they dealt with risk. In this section we go into more detail about the components of COVID labour which we identified in our analysis.

### Seeking and interpreting information

This refers to how participants attempted to understand what was happening in the pandemic, locally, nationally, and sometimes internationally; the evidence around various defence measures; and developments in government guidelines. The latter required the least ‘labour’, as guidelines could be fairly easily accessed, though not always easily understood or followed, as we discuss below. In the first weeks of lockdown, participants reported new rituals in which they watched the daily update from Downing Street on television, often live, to understand how the pandemic was developing and the UK response to it. Their accounts elucidate the many and often difficult attempts participants made to understand what was happening and how best to respond:

I began reading medical reports and studies early on (using my access to online journals in university libraries), relating to pregnancy and covid. There was little info on it, but I saw that there was some question about whether premature labour was linked to pregnant women getting covid. I now read under the headlines of only very select pieces of news, and often in the guardian. I do read things that I think will help me act in a way that would protect us (like stuff about facemasks or social distancing etc). (Acacia Mum [Household Income per annum (HI) £30,000–£59,000, White English,^10^ Parent of a 2-year-old child] June 2020 [Pregnant at the time])

I do use facemasks - disposable as well as re-usable. Started as soon as lockdown started when I was going grocery shopping; on bus and in line at shops/inside shop. Intend to start making own as they are quite expensive and I use quite a lot of them . . . I have looked at the evidence. The homemade one, depending on the fabric you are using, yes they help you a bit, it would help if the other person uses it too, plus social distancing. That is why I have ordered some ‘FFP2’ – masks. (Heather Mum [HI <£30,000, Romanian, Parent of two, 3 and 5 years old] June 2020)

If there is anything important my wife message me that this is the things the changing the lifting the things. She always on the Facebook or WhatsApp messaging with her friend and things and if she gets something important she just text me WhatsApp me I get it from here mostly. (Ilama Dad [HI <£30,000, Bangladeshi, Parent of two, 14 and 16 years old] June 2020)

**Image 1.**
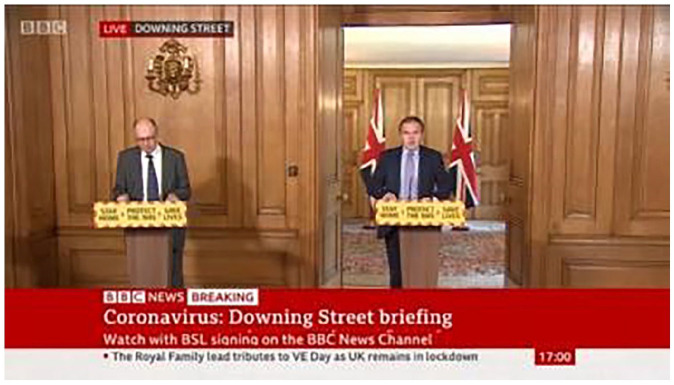
Screenshot submitted by Allium Mum (HI >£120,000, White English, Parent of 2, 10 and 8 years old) May 2020, in response to a prompt from researchers asking about how participants get information about the pandemic. She uploaded this photo and the following accompanying caption: *This is today’s government update – I was watching these avidly at the beginning (I have the BBC news app and a notification let’s me know it’s on) however, for the last few weeks I’ve been less bothered about these. I always want to know the infection rate and death rate for the day. I normally find this on the bbc news app or by watching the bbc news at 10. . . . I don’t watch [government updates] with the kids - not because I’m worried about what they’ll hear / think but just because they’ll be bored by it and I won’t be able to listen in peace! Also, there probably is a bit of me that is sheltering the kids from the news.*

Children’s consumption and exposure was managed by parents (see Image 1), and parents reported seeking guidance around how best to communicate about the pandemic to children. Very few men (only three out of 20) described seeking and interpreting evidence. Like Ilama Dad, men seemed to rely on their partners to relay information, though they were not passively receiving this information. For example, Zenobia Dad (HI >£120,000, Indian) would double check information he heard from relatives that he thought was dubious.

As the weeks went by, more fatigue was apparent in participants’ accounts of seeking information (see also Ogueji et al., 2021), and some reported that they were less steadfast in this task:

I understand the 2m rule, face masks, unlimited exercise. But I am finding it harder to keep up with the latest rule on how many people can meet up and where. (Jasmine Mum [HI £60,000–£89,000, British Mixed Race, Parent of a 2-year-old] September 2020)

Participants also told us that the constant updates on infections and deaths, closely followed initially, became increasingly depressing, as Magnolia Mum (HI >£120,000, Chinese, Parent of a 4-year-old) describes:

Since about Apr[il],^11^ I don’t really focus too much on the Covid statistics anymore, otherwise I feel too negative about things . . . I normally read the headlines on BBC, but I don’t bother reading the details. (June 2020)

Magnolia tells us that she avoids COVID statistics, which leave her feeling ‘too negative’, signalling the emotional burden that could be experienced in following news and updates. Others reported feeling de-sensitised as time went on. Rules were investigated then on a more ad hoc basis – for example, in responding to a social invitation. More complex or contextualised guidelines created more work, and participants in Scotland, Northern Ireland and Wales reported more effort being needed to understand localised restrictions when media sources often focused on England.

Nonetheless, several participants described extensive effort to do research on appropriate social-distancing measures and vaccinations throughout the data collection period. As predicted by Beck (1992) in his writing on ‘Risk Society’, lack of trust in the government was a notable driver here, as participants felt it necessary to go beyond the official guidelines, thereby increasing the personal responsibility felt in looking for and interpreting public health guidance. For example, Daffodil Grandmother (Retired, White), who initially expressed confidence in Prime Minister Johnson and the UK approach, began to lose faith as numbers of COVID deaths and infections rose. She wrote:

Now I am beginning to lose faith in the government and particularly Boris, who seems to have returned to his old bumbling self . . . and today he found Keir Starmer difficult to deal with at PMQT.^12^ Consequently I am beginning to be uncertain about what we can or cannot do. (June 2020)

Similarly, Clover Mum (HI £90,000–£119,999 a year, White, Parent of a 4-year-old) wrote about how she lost trust in the UK approach after the Chief Advisor to the Prime Minister, Dominic Cummings, was widely thought to have broken social distancing rules.^13^ She wrote in late May 2020:

I’m not going to be more reckless in my approach to the ‘guidelines’ as a result. But in my eyes this government has lost any authority – scientific, moral or otherwise – to tell me what to do. I’d like to think I’ll continue to use good judgement but I will not listen to them.

Her distrust prompts her to do her own investigations reviewing scientific literature and guidance from other countries. Similar findings were uncovered in the Netherlands, where Bröer et al. (2021) argued that the gradual easing and later reinstating of restrictions provoked uncertainties around the usefulness and effectiveness of various measures. As the risk of catching or dying from the virus felt more remote, risk and uncertainty shifted to the *management* of the response and less the actual risk of catching COVID. Our findings concur with previous research which shows that those with lower trust in media and governmental institutions are less likely to abide by recommended interventions (Prati et al., 2011; Rönnerstrand, 2013). Clover Mum, however, goes *above and beyond* the UK social distancing guidelines, avoiding shops and restaurants even after they are reopened. She draws on the skills and resources accrued within her job as a civil servant and her educational background, which give her access to the most up-to-date research. She iterates the various resources which she accesses and compares their findings to the UK government recommendations. As the vaccination rollout advanced in the spring of 2021, we saw a similar pattern: those who reported a general trust and/or knowledge of science (via their educational or occupational background) reported their confidence in the vaccine rollout. Jasmine Mum (HI £60,000–£89,000, British Mixed Race, Parent of a 2-year-old, June 2021) for example told us ‘I trust that they’re doing rigorous trials and all that’ in response to a question about any doubts around taking a vaccine. Those with a more sceptical view on science reported ‘waiting and seeing’ and comparing reports on vaccines from different sources.

Some of our participants on lower incomes had no computer and limited internet access, making individual research more problematic. This in turn made them more reliant on social networks for information. This reflects previous research which shows that varying levels of internet access and skills influence the benefits that can be accrued from communication technologies (e.g. Dimaggio et al., 2004). Until now, most attention has been paid to the impact of such inequalities on children’s learning during the pandemic.

### Assessing risk

This second feature of COVID labour refers to how participants make everyday assessments about whether and how they should go about what were previously perceived as commonplace activities. In these accounts, participants revealed how even the most banal activities now involved protracted deliberations and (re)assessments about the correct course of action. Here Kalmia Grandmother, for example, reflects on an encounter she had on a pavement early in the pandemic:

On the pavement, I was waiting for a woman in a wheelchair to signal which way she intended to go. There was no room on the pavement for me to pass her and she had paused at right angles to me. I think perhaps she was waiting for me to turn around and walk back to a wider bit of pavement but I hadn’t realised this. I thought she was examining the display of plants near her. She turned the wheelchair swiftly and passed close to me coughing as she went. I was very shocked and tried a combination of stepping aside into the road and looking back at her in shock. (Kalmia Grandmother, June 2020)

It is clear in this account that Kalmia Grandmother is not sure what is the appropriate behaviour, and that she is fearful of being seen as rude, but also of going too close to the other person. The interaction is ultimately an unpleasant one, and she feels she has missed important cues for appropriate behaviour. While fleeting and perhaps trivial, it clearly remained with Kalmia Grandmother sufficiently for her to relate it to us several weeks later. This interaction, like others, illustrates the in-the-moment assessments of various kinds of risk which individuals negotiated. Other participants described their deliberations around the necessity of shopping visits, as well as where was perceived as safe and who in the household should go. These deliberations in the initial days were about how to navigate within the guidance, such as the one trip to the shops per day and no mixing across households:

[Son’s] new computer is delivered. We discuss whether it should be left at the door but agree to allow our friend, who built it to come in and set it up. We all wash our hands afterwards although there has been no physical contact. (Daffodil Mum [HI £30,000–£59,999, White English, Parent of three, 12, 14 and 16 years old] May 2020)

We’d decided that my wife would do most of the going out as she was still going to work (on a rota) as she’s a teaching assistant and the school is still open looking after the children of key workers. It seemed that it reduced the risk of bringing Coronavirus into the house to only one person. I’m not sure if that logic stands up to close scrutiny but if you don’t look too hard it sort of makes sense. (Daffodil Dad [White English] May 2020)

For single parents, such divisions of tasks were not possible, and single parents with young children in particular reported the difficulties they had in weighing different risks around buying groceries:

But yeah it is a worry and where shopping did take much longer, waiting in the queues whatever, yeah I was anxious but I was like ‘do I leave them at home where they’re safer, or do I take them out with me where it’s not so safe?’. There’s a couple of times I took them with me and I left them in the car when I went into the shop, but that was like ‘ok how long can I leave them for, they haven’t got a phone if I need to contact them’. There was a lot of anxiety behind that, there’s so many issues. (Mallow Mum [HI £30,000–£59,999, British Asian, Parent of three, 11, 10 and 6 years old] June 2020, Interview)

A key issue which parents discussed with us was about keeping their young children in school or nursery care (the latter is not compulsory for children under the age of five in the UK and the former was optional for the families of key workers):

We have had different opinions on going back to school, although we have been talking about it a lot, obviously, and eventually we came to an agreement which we are okay with, and we’ve got benchmarks for when we wouldn’t be happy with continuing that option. Xylosma Mum [HI >£120,000, White English, Parent of a 5-year-old] June 2020)

The labour involved here is clear – protracted discussions and even agreed benchmarks as they move forward with their agreed position – demonstrating the relational nature of assessing risk as participants negotiated within and across households. The Kalmia family, on the other hand, were apparently overruled by the father, who preferred to take the children out of school early, before lockdown began, against the preferences of his wife and children. Here consultation broke down. This is a kind of labour too, in having to deal with the repercussions where there remains some disquiet about the decision that was made, even 10 weeks later when Kalmia Mum tells us about it.

**Image 2.**
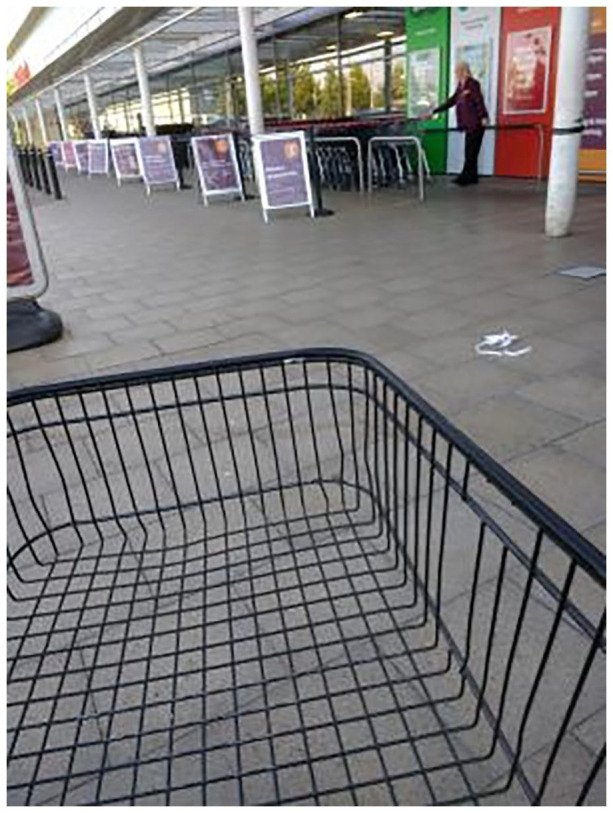
Photo uploaded by Orchid Grandmother (Retired, White Scottish) 1 June 2020, in response to a prompt from researchers asking participants to describe their daily life. She writes about this photo: *Sunday morning at Sainsbury’s - no queue. Unfortunately I also wanted to collect an Argos order from the in-store area and the number of people waiting there was huge so that plan was quickly abandoned.*

As the initial lockdown eased, risk assessments became more commonplace as the parameters for behaviour widened (see Image 2). Here Echinacea Mum (HI £60,000–£89,999, White English, Parent of a 7-year-old) recounts a discussion with her father about going to a restaurant in late June 2020:

[I had ] a discussion with my dad on whether we should go to the Balti and eat out, we were concerned that other people may not stick to guidelines especially after a few drinks. We were also concerned that the Balti wouldn’t encourage social distancing measures so could potentially spread the virus i.e. someone shakes their hand which usually happens, they are very nice at the Balti and I don’t think they would want to offend. We decided it wasn’t worth the risk so didn’t go.

Perhaps inevitably, those who identified particular vulnerabilities to COVID, such as via underlying conditions or older age, were most risk averse. Echinacea Mum was worried about exposing her elderly father to the virus, with whom she lived, for example. Parents who had immigrated to the UK as adults or others living far from wider networks also expressed heightened anxieties around catching COVID, explaining their worries about who would look after their child(ren) should they get ill or die. (In line with the third category, ‘minimising risk’, this led Zenobia Mum and Dad to sterilise all groceries for the full year of the study, for example.) In general, minority ethnic participants commented on their heightened risk of catching COVID as reported in the media, and the anxieties which this produced for them. Others discussed how they balanced risk of exposure to the virus, with other priorities in making life ‘livable’, usually in relation to the perceived necessity to see friends and family:

So, I felt, you know, [at Christmas] I’d had it with lockdown and I couldn’t see him [brother who is very ill] and I thought ‘I’m not going to observe the rules because it’s more important to see somebody before they die’. (Kalmia Grandmother [Retired, White English] June 2021)

Assessing risk then involved considering multiple kinds of perceived risks, which were often contradictory. Kalmia Grandmother expresses a moral authority in her decision-making, which apparently eased the dilemma, though her retrospective account is also coloured by the fact that (to her knowledge) the visit did not ultimately result in illness or death. In other instances, there was much more anguish expressed, as can be seen here with Begonia Mum (HI £30,000–£59,000, White English, Parent of three, 4, 7 and 9 years old) when she discusses what happened when her daughter had a temperature after she started at school in person in October 2021:

I had a sleepless night agonising about what to do. I was 99.9% certain that it wasn’t Covid as it’s characteristic of my daughter to hit the wall a couple of weeks into school and her usual response is to conk out for 24 hours. I kept her off school for a day as a precaution but did not go as far as testing her and isolating us/my other children. It was the right call but I suppose it might not have been. In the absence of fast testing I certainly feel huge pressure in having to make those judgement calls.

Begonia Dad did not comment on this episode; in this household the mother took on the majority of care and household work, which ultimately meant more COVID labour for her too.

These examples show that even in periods of tight control, participants were involved in multiple and sometimes daily decisions around risk. Such assessments could be small and immediate, or protracted and repeated. In most cases, participants were faced with decisions where no outcome was desirable – e.g. forgoing the opportunity to visit a dying brother or going but taking into account that you may put the lives of others in danger. These accounts underline how risk assessment can be experienced as a kind of suffering in and of itself – see the repetition of ‘agonising’ for example in the accounts above. Jane Elliott (2013) analyses castaway and survival genres for how they ‘make manifest’ the experiences of ‘suffering agency’ (p. 93) which she sees as inherent in neoliberal governance. This suffering, she says, unfolds at the ‘intersection of interest, choice, and agential action’ (p. 84). Our participants, we argue, are caught in this configuration as they grapple between risks associated with catching and/or transmitting COVID-19, particular understandings of responsibility to intimate (and non-intimate) others, and their own needs in creating and sustaining a livable life. The *suffering* which Elliott refers to is in the agency or responsibility of the individual in having to decide between two bad options. Elliott demonstrates how agency, often thought of as necessarily ‘good’, can also be experienced as oppressive. Such suffering is heightened in those who understood themselves as at greater risk of catching and dying from the virus (including minority ethnic participants) and those on lower incomes (more discussion on this below), and was more often spoken of by mothers who, as we know from other research, are more likely to take responsibility for risk avoidance within families (Mackendrick, 2014). These accounts illustrate the personal responsibility to respond appropriately to public health measures which individuals grapple with during a pandemic, and how they are mediated by their relationships with others. They also highlight how the loosening of restrictions could paradoxically be experienced as more anxiety-provoking, as participants were faced with often difficult decisions on how to respond to guidance.

### Minimising risk

In this section, we describe the practical strategies enacted by participants to avoid catching and/or transmitting the COVID-19 virus to others. Those who felt most vulnerable to the virus, for various reasons noted above, enacted the most processes for avoiding risk. These ‘stricter’ families often avoided any social contact with other people or in public spaces:

I have not used and as yet do not have any face masks. In truth, I have barely been out in any indoor public buildings since mid-March. Because of having a child with severe disability, as a family we have mostly isolated ourselves and have only been on outdoor walks mostly in the rural area around where we live. I think we are going to struggle with feeling safe enough to resume normal life. (Bacopa Mum [HI £30,000–£59,999, White Irish, Parent of three, 15, 12 and 10 years old] June 2021)

I haven’t been shopping for 4 months now. I can’t go at all because if I go, I have to take him [8 year old son with autism] and there is no way I can say don’t touch this and that, so. We are like a prisoner in our own house at the moment. (Holly Mum [HI <£30,000, Nepali, Parent of 9 year old] July 2020)

Participants from lower socio-economic groups had least recourse to this strategy of total or near-total isolation. These participants were more likely to work in jobs which demanded on-site presence and more frequently described leaving the house to use public spaces for leisure when they didn’t have a garden or even much space in their homes (see also Rosenthal et al., 2020). Wealthier families could sign up to supermarket deliveries or farmers’ market boxes brought directly to their door, while lower income families were reliant on frequent visits to shops for a small number of items, often in search of more affordable options. Elderberry Mum (Black African, HI <£30,000, Parent of three, 12 and 5 years and 5 months old), for example, discussed her difficulties in accessing formula milk for her newborn baby. As a single mother of three children on universal credit she was sometimes reliant on food banks, which have a policy of not supplying formula milk (to encourage breastfeeding). She reports being advised against leaving her home with her son (aged 7) who was deemed vulnerable to COVID-19 as an asthma sufferer, and more than once had COVID-like symptoms. Here she describes her reaction to the medical advice to not let him leave the house:

She [medical doctor] told us not to take him out again, I said I don’t take him out, and she said I shouldn’t go out. I’m like I shouldn’t go out then how do I feed my boys?? (June 2020)

Ultimately, she took him with her as she went from shop to shop to find affordable formula milk. Elderberry Mum’s lack of financial resources was further compounded by her lack of social support, meaning there was no one she could turn to for help in caring for her children while visiting shops. This example demonstrates the extra difficulties faced by families on lower incomes, as well as the unworkability of guidance to ‘stay home’ which was issued for those self-isolating or shielding.

Second to household isolation was relying on various resources in mitigating the risks posed by the virus and in responding to government mandates about, for example, staying home or wearing facemasks. As discussed above, families required digital software and know-how for adults and children alike to access public health information, social networks, work and education. Leaving the home required facemasks and hand gel, which were not always easy to come by. In May 2020, for example, there was a national shortage in antibacterial hand gel, with several shops limiting the number of bottles available to each consumer and some hiking up the prices as demand increased. The labour involved in obtaining and managing these resources required to maintain one’s safety and wellbeing are illustrated well in the case of the Nectarine family. Nectarine Dad is an Uber driver of Black African origin with a household income of less than £30,000. His youngest child is on the shielding list, but as they have only a very limited income from his wife, who works 3–4 hours/week, he must continue with this relatively high-risk job. He purchases masks, wipes, anti-bacterial spray for the car and hand gel. He also changes and washes his clothes daily as he enters from work in a bid to reduce the risk of infection to his wife and daughter. Those in lower status jobs, such as Nectarine Dad, need to employ *more* resources to avoid infection than those who have higher status jobs that can be undertaken at home, or other key workers such as medics, who (largely speaking) were provided with protective equipment by their employers. Poorer households, whose earnings were likely to have been impacted the most during the pandemic (Bourquin et al., 2020), expended a greater proportion of their income on these necessities than those with higher economic resources. Ironically, those on higher salaries are likely to have actually saved money during the pandemic, as less of their earnings were spent on recreational activities, commuting and private childcare.

These defence items also held symbolic value for participants, and their use could prove contentious. Magnolia Mum, who is of Chinese origin, reported how she was reluctant to be seen in a mask before they were mandatory fearing racist retribution from those around her. Lavender Mum had a medical reason not to use masks, but nonetheless sometimes wore them to avoid microaggressions from members of the public scolding her (see Lupton et al., 2021 for similar findings). Both of these constitute a particular form of cognitive labour in dealing with the consequences of using and displaying (or not) various pandemic defence resources. The rollout of the vaccine was similarly moralised and often divisive. ‘Pro-vaccine’ participants described their incredulity of those who were vaccine hesitant, while several vaccine-hesitant participants reported keeping their opinions about the vaccines to themselves for fear of reprimand. For some participants, different opinions around vaccinations were too much to bear, reporting that such differences could not be overcome and ultimately some social ties were cut (see Twamley et al., Forthcoming 2023, for more detail). As argued by Reicher et al. (2021), such charged divisions and recriminations around various risk mitigating measures are likely to have been exacerbated by the government’s continual focus on individuals as not sufficiently or correctly following public health recommendations, and the characterisation of such individuals as ‘covidiots’ or ‘selfish’.

## Conclusion

Our detailed ethnographic study of life under lockdown for families with children in the UK, reveals the everyday labour which is involved in adapting and responding to a global pandemic. Drawing on scholarship around the sociology of everyday life (Back, 2015; Neal & Murji, 2015), cognitive labour (Daminger, 2019) and ‘risk work’ (Brown & Gale, 2018; Gale et al., 2016), we describe how adults in families experience and negotiate the risks encountered during COVID-19. We call this COVID labour, entailing three main aspects – seeking and interpreting information; assessing risk; and minimising risk. These overlap with, but are necessarily different from, the constituent parts of ‘risk work’ developed by Brown and Gale (2018; see also Gale et al., 2016) in their studies of healthcare professionals’ experiences of managing risk in their everyday work. Participants in our study were parents and grandparents managing risk during an unprecedented transformation of everyday life. They were often juggling paid and unpaid work when childcare institutions and other support systems were no longer available. Our work on COVID labour highlights another form of labour which anyone with caring responsibilities was likely to have experienced during this time. This work was tiring and emotionally challenging: our participants discussed their confusion, anxiety and anguish as they struggled to understand and respond to the pandemic situation, not least because this is a highly moralised issue for parents as it relates to keeping children safe.

Such labour was apparent, though in different ways, across the period of data collection – from May 2020 to June 2021. In the earlier part of the pandemic, most energy was expended in understanding social distancing guidance. Later, as various public health measures were relaxed, there was an apparent increase in assessments of risk, as government guidance was more open to interpretation. As predicted by Beck (1992), distrust in certain institutions did give rise to an increased sense of personal responsibility in responding to risks. For example, those that had least trust in the government’s response to the pandemic, undertook the most labour in uncovering and interpreting ‘trustworthy’ information, and in consulting with others as they sought to appropriately protect themselves and others. Likewise, those who felt most vulnerable to the virus made greater efforts to minimise exposure and did so for longer lengths of time. However, it was clear that those on lower incomes were least able to minimise risk, laying bare the inadequacies of ‘stay at home’ measures which make too many assumptions about the kinds of resources which individuals can draw on in attending to social distancing guidelines (see also Preston & Firth, 2020). This should be useful for policy makers as they consider the context in which ‘compliance’ to social distancing and other measures are negotiated.

Our findings may also point to some of the mechanisms potentially underlying poorer mental health outcomes reported amongst women, those on lower incomes and those from minority ethnic backgrounds (Banks & Xu, 2020). The heightened levels of COVID labour which these individuals experience is a cognitive burden, akin to the ‘mental load’ uncovered in other studies (Mackendrick, 2014). Such labour can have significant psychological and behavioural consequences (Mullainathan & Shafir, 2013; Vohs et al., 2008). We argue that the very agential aspect of COVID labour – in particular around risk assessment – is experienced as ‘suffering agency’, as participants attempt to deliberate between equally unappealing and sometimes life-threatening options. This suffering is likely exacerbated by processes of individualisation and UK government emphases on individual culpability in any failures of COVID public health measures (Reicher et al., 2021). Indeed, if anything, what this study demonstrates is that this COVID labour is always relational and negotiated both within and across families, as well as being stratified along familiar lines of gender, ethnicity and class. As such, it provides a sociological context for policy making that not only aids our understanding of contemporary events, but that should inform the management of future pandemics.

## Notes

1. See timeline of UK lockdowns and restrictions here: www.instituteforgovernment.org.uk/charts/uk-government-coronavirus-lockdowns

2. We use the term ‘COVID labour’ over ‘COVID work’ in reflecting the common scholarly distinction between unpaid labour and paid work.

3. Gov.uk. 2020 Guidance: Making a support bubble with another household. www.gov.uk/guidance/making-a-support-bubble-with-another-household

4. See: www.police.uk/tua/tell-us-about/c19/v7/tell-us-about-a-possible-breach-of-coronavirus-covid-19-measures/

5. One household contained non-family members (as defined by the participants): ‘lodgers’ living with Quince mother and daughter. The lodgers did not participate in the study.

6. Average household income of two parent household with two children is approximately £40,000 a year. See: https://ifs.org.uk/tools_and_resources/where_do_you_fit_in

7. As defined in the first national lockdown – working in essential services that required in-person location at work.

8. Currently 50% of school-leavers attend university, thus our sample over-represents higher educated individuals.

9. In some cases only one member of a family participated, meaning both the first and final interviews were individual interviews. Three families never completed a final interview – Bacopa, Katsure and Narcissus.

10. Here we refer to racialised category (‘white’ or ‘black’ for example) and also nationality which we break down to the four nations of the UK, for those born and/or brought up in the UK. All participants from Northern Ireland, Scotland and Wales identified as white and born in the UK.

11. In general text submitted by participants is presented verbatim, but on occasion we have edited for clarity.

12. Prime Minister’s Question Time.

13. See: www.bbc.co.uk/news/uk-politics-52784290
